# Differential diagnosis of progressive intellectual and neurological deterioration in children

**DOI:** 10.1111/dmcn.14691

**Published:** 2020-09-24

**Authors:** Christopher Verity, Elaine Baker, Polly Maunder, Suvankar Pal, Anne Marie Winstone

**Affiliations:** ^1^ The PIND Research Group Addenbrooke’s Hospital Cambridge UK; ^2^ The National Creutzfeldt‐Jakob Disease Research and Surveillance Unit University of Edinburgh Edinburgh UK

## Abstract

**Aim:**

To report the differential diagnosis in children with progressive intellectual and neurological deterioration (PIND) in the UK.

**Method:**

Since 1997 the PIND Study has searched for variant Creutzfeldt‐Jakob disease (vCJD) in children, using the British Paediatric Surveillance Unit to perform prospective surveillance of those younger than 16 years with PIND.

**Results:**

From May 1997 to October 2019, 2255 children meeting PIND criteria had been notified, of whom 2008 (1085 males, 923 females) had underlying diagnoses. There were over 220 different diseases, including six cases of vCJD. The numbers presenting in four age groups were: <1 year, 805 (40%); 1 to 4 years inclusive, 825 (41%); 5 to 9 years inclusive, 264 (13%); and 10 to 15 years inclusive, 114 (6%). The two largest ethnic groups were White and Pakistani (58.2% and 17% of diagnosed cases). The most common diseases in these two ethnic groups are shown for the four age groups. The distribution of diseases varied with age but was quite similar in White and Pakistani children.

**Interpretation:**

This paper provides a unique guide to the complex differential diagnosis of childhood PIND, showing considerable differences between four age groups, but similarities between ethnic groups. The PIND Study still provides the only systematic surveillance for vCJD in children in the UK.

**What this paper adds:**

- The prevalence of diseases causing childhood progressive intellectual and neurological deterioration in the UK is low (approximately 0.1/1000 live births).
- There were more than 220 different disorders, mainly genetically determined.
- The majority of disorders presented early in childhood: 81% before the age of 5 years.
- There were similarities in the disease spectrum in White and Pakistani children.

## What this paper adds

- The prevalence of diseases causing childhood progressive intellectual and neurological deterioration in the UK is low (approximately 0.1/1000 live births).
- There were more than 220 different disorders, mainly genetically determined.
- The majority of disorders presented early in childhood: 81% before the age of 5 years.
- There were similarities in the disease spectrum in White and Pakistani children.

Video Podcast: https://youtu.be/QbUG-ZVJLHc

This article is commented on by van Karnebeek on page 243 of this issue.

**Abbreviations**
BPSU: British Paediatric Surveillance Unit
NCJDRSU: National Creutzfeldt‐Jakob disease Research and Surveillance Unit
PIND: Progressive intellectual and neurological deterioration
vCJD: Variant Creutzfeldt‐Jakob disease

The first cases of variant Creutzfeldt‐Jakob disease (vCJD) in young adults were reported by the National Creutzfeldt‐Jakob Disease Research and Surveillance Unit (NCJDRSU) in 1996.^1^ This raised the possibility that vCJD might occur in children. As there is no validated screening test for vCJD it is necessary to search systematically for cases of vCJD among the many neurodegenerative disorders of childhood. That is why the study of progressive intellectual and neurological deterioration (PIND) was set up in 1997, using the well‐established system pioneered by the British Paediatric Surveillance Unit (BPSU).^2^, ^3^ Paediatricians in the UK are asked to notify all patients aged younger than 16 years with PIND because this group of children is likely to include any cases of vCJD. There is still a risk that children could develop vCJD, so PIND surveillance continues.^4^

Most of the notified children who meet the study criteria for PIND have an underlying diagnosis other than vCJD to explain their deterioration, and an independent PIND Study Expert Group validates those diagnoses. Since 1997, the PIND Study has provided a unique, on‐going review of the distribution of rare neurodegenerative diseases in children in the UK. The study has previously reported that some UK paediatric centres notify relatively large numbers of cases with a high proportion of children of Pakistani origin.^4^, ^5^ This paper provides clinicians with an age‐related differential diagnosis in children with PIND, concentrating on the two largest ethnic groups in the PIND Study (White and Pakistani).

## Method

Since 1986, the BPSU has studied many rare childhood disorders. All UK consultant paediatricians are requested to notify children with one of the conditions under surveillance using a monthly online system asking for notifications of all children seen in that month who meet the case definition for PIND (Table 1), even if a diagnosis has already been made (see the BPSU section of the Royal College of Paediatrics and Child Health website).^6^

**Table 1:** Case definition – progressive intellectual and neurological deterioration

Any child (under 16y of age at onset of symptoms) who fulfils all of the following three criteria: Progressive deterioration for more than 3mo; Loss of already attained intellectual or developmental abilities; and Development of abnormal neurological signs
Excluding
Static intellectual loss (e.g. after encephalitis, head injury, or near drowning)
Including
Children who meet the case definition, even if specific neurological diagnoses have been made
Metabolic disorders leading to neurological deterioration
Seizure disorders if associated with progressive deterioration
Children who have been diagnosed as having neurodegenerative disorders who have not yet developed symptoms
Reporting restricted to cases seen in the last month but including those whose condition began earlier (i.e. including ‘old cases’ of children in follow‐up if seen in that month)

Clinical information is obtained via a questionnaire completed by the notifying paediatrician, via telephone interview or by site visit; there is no contact with patients or families. The questionnaire records information about presenting symptoms and signs and follows progress after presentation. It also enquires about the child’s ethnic origin, using the classification that was used by the Public Health Laboratory Service in 1997: White, Black Caribbean, Black African, Black other, Indian, Pakistani, Bangladeshi, Chinese, Other. The clinical data are anonymized and then reviewed by an independent PIND Expert Group to confirm and classify diagnoses. The Group consists of paediatric neurologists, specialists in genetics and paediatric metabolic disease, together with representatives from the NCJDRSU. If vCJD is suspected it is suggested that the local paediatrician obtains consent to refer the child to the NCJDRSU in Edinburgh. Otherwise, the notified children are followed up by the PIND team via the local clinician until: (1) a definite diagnosis is made, (2) no further investigations are planned, or (3) the child dies.

As recommended by Foss et al.,^7^ the lifetime risk at birth was calculated for the cases of confirmed PIND by dividing the number of diagnosed cases by the number of total births during the relevant diagnosis period.

The PIND Study was approved by the National Research Ethics Committee East of England Cambridge Central (ref 97/010). The Public Health England Caldicott Advisory Panel has agreed that patient identifiable information can be processed without consent under Regulation 3 of the Health Service (Control of Patient Information) Regulations 2002. The Public Benefit and Privacy Panel for Health and Social Care (Scotland) has similarly approved the PIND Study.

## Results

Between May 1997 and October 2019, 4612 children had been notified to the PIND Study. After an initially large number of notifications of prevalent cases in the first 20 months of the study, the number of notified cases of suspected PIND settled over the period 1999 to 2018 inclusive to between 146 and 230 per year (median 188.5/year); this notification rate has continued to be relatively constant. The number of confirmed cases of PIND that had been fully investigated was 2255; 2073 cases were not included (1023 cases did not meet the PIND criteria, there were reporting errors/duplicate notifications in 786, and clinical details were not forthcoming in 264 cases). A further 284 were either still under investigation or clinical details were pending. The lifetime risk at birth of having a disease causing PIND was calculated using the 1703 diagnosed cases meeting the PIND criteria who were notified between the beginning of 1999 and the end of 2018, when numbers had settled to a relatively steady level of incident cases. During that period there were 14 935 076 live births in the UK,^8^ so the lifetime risk of having a disease causing PIND was 0.1 per 1000 live births.

Of the 2255 children notified between 1997 and 2019 who met PIND criteria and had been investigated, 247 did not have an underlying diagnosis to explain their deterioration: all investigations had been negative, or they had died undiagnosed. That left 2008 (1085 males, 923 females) with a known underlying diagnosis, with over 220 different disorders including six cases of vCJD. In these 2008 cases the age at presentation (defined as the age at which the child first developed symptoms or clinical signs) was determined from the clinical records. On this basis the cases were allocated to four different age groups: <1 year: 805 (40%); 1 to 4 years inclusive: 825 (41%); 5 to 9 years inclusive: 264 (13%); 10 to 15 years inclusive: 114 (6%); thus 81% presented before 5 years of age. The distribution by ethnic origin was: White 58.2%, Black 1.9%, Indian 1.3%, Pakistani 17%, Bangladeshi 2.2%, Chinese 0.25%, Other (other ethnic groups, mixed race, unknown, etc.) 19%. Of the 340 children in the Pakistani group, 294 (86%) had consanguineous parents and 17 (5%) did not; in 29 (9%) that information was not available. In contrast, only 3% of the 1169 White children had consanguineous parents, although information about consanguinity was not available for 21% of the White children.

**Figure 1:**
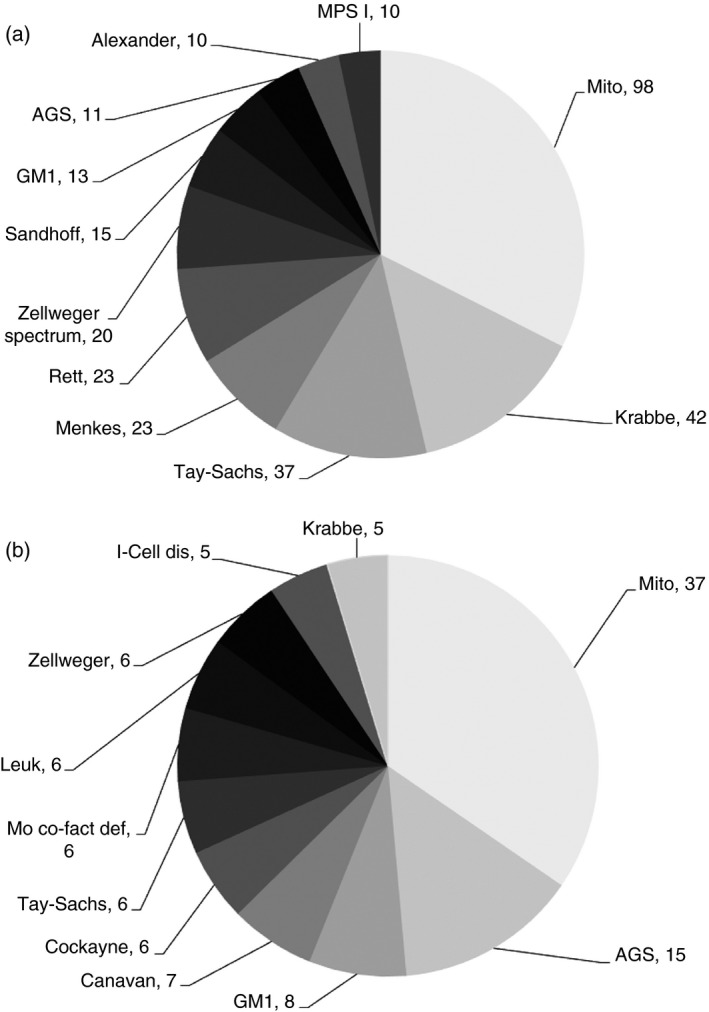
rogressive intellectual and neurological deterioration diagnoses in (a) White and (b) Pakistani children <1y. Eleven most common diagnoses in White (*n*=302) and Pakistani (*n*=107) children age <1y. The White (*n*=150) and Pakistani (*n*=62) children with ‘other’ diagnoses are shown in Table S1. AGS, Aicardi‐Goutières syndrome; GM1, GM1 gangliosidosis; Leuk, unclassified leukoencephalopathies; Mito, mitochondrial disorders; Mo co‐fact def, molybdenum co‐factor deficiency; MPS I, mucopolysaccharidosis type I; Zellweger spectrum, Zellweger spectrum disorder (includes Zellweger syndrome, neonatal adrenoleukodystrophy, and infantile Refsum disease).

**Figure 2:**
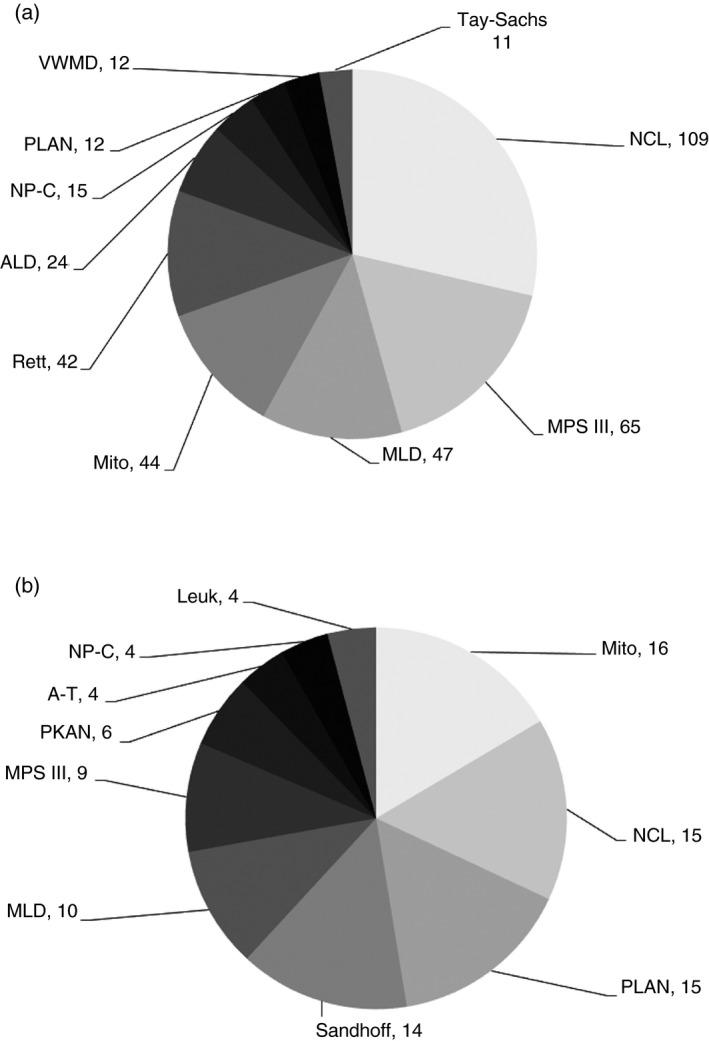
Progressive intellectual and neurological deterioration diagnoses in (a) White and (b) Pakistani children 1–4y. Ten most common diagnoses in White (*n*=381) and Pakistani (*n*=97) children age 1–4y. The White (*n*=96) and Pakistani (*n*=37) children with ‘other’ diagnoses are shown in Table S2. ALD, adrenoleukodystrophy; A‐T, ataxia‐telangiectasia; Leuk, unclassified leukoencephalopathies; Mito, mitochondrial disorders; MLD, metachromatic leukodystrophy; MPS III, mucopolysaccharidosis type III; NCL, neuronal ceroid lipofuscinoses; NP‐C, Niemann‐Pick type C; PKAN, pantothenate kinase‐associated neurodegeneration; PLAN, PLA2G6‐associated neurodegeneration; VWMD, vanishing white matter disease.

**Figure 3:**
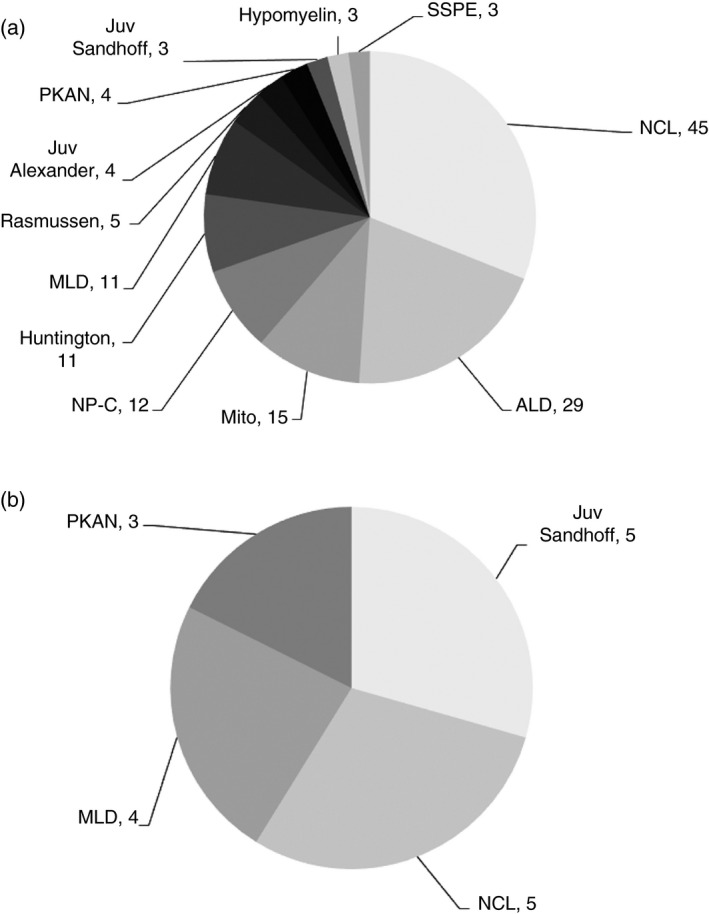
Progressive intellectual and neurological deterioration diagnoses in (a) White and (b) Pakistani children 5–9y. Twelve most common diagnoses in White (*n*=145) children age 5–9y and four most common diagnoses in Pakistani (*n*=17) children age 5–9y. The White (*n*=18) and Pakistani (*n*=10) children with ‘other’ diagnoses are shown in Table S3. ALD, adrenoleukodystrophy; Hypomyelin, hypomyelination; Juv, juvenile; Mito, mitochondrial disorders; MLD, metachromatic leukodystrophy; NCL, neuronal ceroid lipofuscinoses; NP‐C, Niemann‐Pick type C; PKAN, pantothenate kinase‐associated neurodegeneration; SSPE, subacute sclerosing panencephalitis.

**Figure 4:**
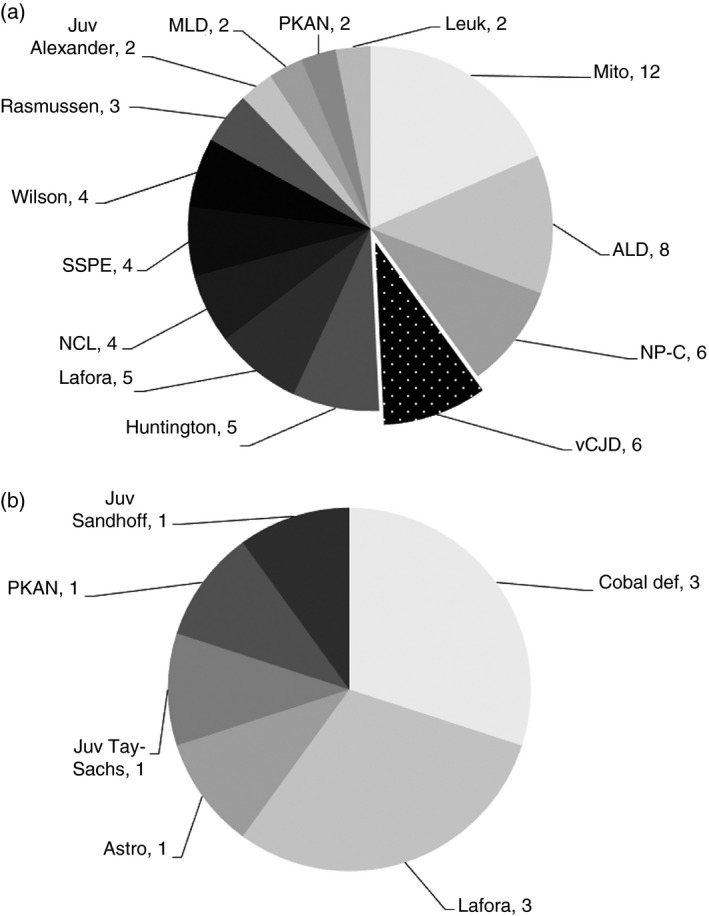
Progressive intellectual and neurological deterioration diagnoses in (a) White and (b) Pakistani children 10–15y. Fourteen most common diagnoses in White (*n*=65) children age 10–15y and six most common diagnoses in Pakistani (*n*=10) children age 10–15y. The White (*n*=12) children with ‘other’ diagnoses are shown in Table S4. ALD, adrenoleukodystrophy; Astro, astrocytoma; Cobal, cobalamin deficiency; Juv, juvenile; Leuk, unclassified leukoencephalopathies; Mito, mitochondrial disorders; MLD, metachromatic leukodystrophy; NCL, neuronal ceroid lipofuscinoses; NP‐C, Niemann‐Pick type C; PKAN, pantothenate kinase‐associated neurodegeneration; SSPE, subacute sclerosing panencephalitis; vCJD, variant Creutzfeldt‐Jakob disease.

The most common of these rare disorders in the two largest ethnic groups (White and Pakistani) in the four age groups given above are shown in Figures 1, 2, 3, 4 and the ‘others’ (those White and Pakistani children who are not shown in the figures) are listed in Tables [Link], [Link], [Link], [Link] (online supporting information). For example, in the <1 year age group there were 452 White children: 302 of these are included in the 11 most common diagnoses shown in Figure 1a and 150 ‘others’ are listed in Table S1. Also in the <1 year group were 169 Pakistani children: 107 of these are included in the 11 most common diagnoses shown in Figure 1b and 62 ‘others’ are listed in Table S1. Similar details about the composition of the other age groups are shown in the legends for Figures 1, 2, 3, 4. The White and Pakistani cases that are not shown in the figures (see also Tables [Link], [Link], [Link], [Link]) had a heterogeneous mixture of diseases. For instance, in the <1 year age group, the White ‘others’ group included 59 diseases and the Pakistani ‘others’ group included 36 diseases. There were 1169 White and 340 Pakistani children, leaving 499 children for whom details about the diagnoses are not included in this paper because (1) the other ethnic groups were so small and (2) the ethnicity of the remainder was not known.

It can be seen in Figures 1, 2, 3, 4 that there are differences in the distribution of diseases according to age. In contrast, there was considerable overlap in the distribution of disease found in White and Pakistani children of a given age. For example, Figure 1a shows the 11 most common diseases in the White children aged <1 year; six of these diseases were also found among the 11 most common diseases in the Pakistani children in the same age group, as shown in Figure 1b. If the diseases that were less common in that Pakistani age group are also considered (see Table S2), then nine of the 11 most common diseases found in the White children were found in Pakistani children of the same age. There were similar findings in other age groups.

For simplicity, some disorders have been grouped. For instance, among White children in the first year of life there were 98 cases with mitochondrial diseases, made up of 21 different mitochondrial diagnoses, also the diseases included in the Zellweger spectrum disorder were grouped together (see Fig. 1a). In White children aged 1 to 4 years there were 109 cases of neuronal ceroid lipofuscinosis, made up of eight different neuronal ceroid lipofuscinosis diagnoses (see Fig. 2a).

White children with mucopolysaccharidosis type III were most likely to present at age 1 to 4 years. A large proportion of children with neurodegeneration with brain iron accumulation were in this age group. There were three different neurodegeneration with brain iron accumulation diagnoses (shown in Fig. 2a,b, and Table S2): pantothenate kinase‐associated neurodegeneration, PLA2G6‐associated neurodegeneration, and beta‐propeller protein‐associated neurodegeneration. Children with Rett syndrome lose skills towards the end of the first year of life and are among the most common PIND cases of White children in the <1 year and the 1 to 4 year age groups. However, older children with Rett syndrome are more stable neurologically and do not meet the study criteria, leading to under‐ascertainment of Rett syndrome cases. In the 5 to 9 year and 10 to 15 year groups, Rasmussen syndrome and Huntington disease appear in White children (there was no case of Huntington disease in the Pakistani group).

The differential diagnosis in the White children in the 10 to 15 year age group includes all six children with vCJD notified to the PIND Study between 1998 and 2001. The youngest was a female who developed symptoms at age 12 years. The clinical features in these children were similar to those in adults with vCJD (neuropsychiatric features, new ataxia, cognitive decline). The investigations that were used to confirm the diagnosis in children were the same as those used in adults (e.g. the pulvinar sign on brain magnetic resonance imaging and post‐mortem neuropathological findings).^3^

It is notable that new cases of subacute sclerosing panencephalitis disappeared for a time. By October 2019, 13 cases of subacute sclerosing panencephalitis had been notified and confirmed. Of these, eight were White children: one in the 1 to 4 year group (Table S2), three in the 5 to 9 year group (Fig. 3a), and four in the 10 to 15 year group (Fig. 4a). One was a Pakistani child in the 5 to 9 year group (Table S3); three were in other ethnic groups, and in one ethnicity was unknown. Of the 13 subacute sclerosing panencephalitis cases, nine had developed symptoms in or before 2002. There was then a gap of 14 years until a child was reported who had developed symptoms in 2017, and three more followed. Subacute sclerosing panencephalitis is a late complication of measles infection, so it seems likely that the recurrence of cases results from falling measles vaccination uptake. The number of confirmed cases of measles (in adults and children) in England and Wales increased from 56 in 1998 to a peak of 2032 in 2012; in 2018 it was 989.^9^

## Discussion

### Background

Until 2016, all the 177 deaths from definite and probable cases of vCJD^10^ had occurred in patients who were methionine homozygous at codon 129 of the *PRNP* gene. However, in January 2017 it was reported that vCJD had been confirmed in a UK male who was methionine/valine heterozygous at *PRNP* codon 129.^11^ In the general UK population, about 44% have the methionine homozygous genotype and 45% are methionine/valine,^12^ raising the possibility of a second wave of vCJD cases in those with the methionine/valine genotype. Also, there are concerns about surgical^13^ and blood‐borne transmission,^14^ so there is a need for continuing national surveillance for vCJD. UK surveillance for prion diseases is carried out by the NCJDRSU, the team that first identified vCJD,^1^ relying on neurologists, psychiatrists, neuropathologists, and other clinical specialists to report adults with suspected vCJD. When planning surveillance in children it was clear that vCJD cases could be hidden among the multitude of rare neurodegenerative diseases of childhood, so a system of direct referral of suspected vCJD cases would not be reliable. Therefore, since 1997 the PIND Study has asked paediatricians to notify all the children in the UK with PIND, looking for vCJD cases among them.

### Strengths of the study

The PIND Study uses the health surveillance mechanism of the BPSU.^2^ There is multisource ascertainment and the reporting is not restricted to cases admitted to hospital. All the cases reported are investigated according to normal practice via the usual networks established by community paediatricians, general paediatricians, and other specialists (e.g. geneticists, paediatric neurologists, metabolic specialists, neuropathologists, and others), so information about cases is available from general paediatrics and sub‐specialists. The PIND Study team makes no contact with the patient or the family and does not become involved in the management of cases.

Notified children are followed via the notifying paediatrician until they are diagnosed, until no further investigations are planned, or until the child dies. It is a strength of the study that all suspected PIND cases are reported, even if many are later excluded because their clinical course does not fit the study criteria. By October 2019, the PIND Study Expert Group had agreed a diagnosis in 2008 confirmed cases of PIND, thus providing an independent validation of more than 220 known neurological disorders. The PIND Study has published case series of some of the diagnosed groups.^15^, ^16^, ^17^, ^18^

The PIND Study protocol stipulates that paediatricians who report children with suspected vCJD are advised to refer those children to the NCJDRSU. All six children in the UK with vCJD were notified to the PIND Study and were also investigated by the NCJDRSU. If the PIND Study had not been in operation these children might not have been ascertained by the NCJDRSU surveillance programme, because it focusses on adults. No new childhood vCJD cases have been identified in the UK since 2001. The last adult vCJD case in the UK was diagnosed in 2016.^11^ The hope is that no new cases of vCJD will occur, but the risk remains.

### Limitations of the study

The BPSU relies on the voluntary support of consultant paediatricians: it does not provide complete ascertainment of cases and it is not always possible to obtain all the relevant data about individual cases. After PIND cases have been diagnosed they are not systematically followed up by the PIND Study and follow‐up has to be discontinued, eventually, in undiagnosed cases, including some cases who are transferred to adult care. It should be born in mind that this study does not provide a guide to all neurodegenerative diseases in children as many do not meet the case definition for PIND.

### Comparison with other studies

There is a very specific case definition for the PIND Study (see Table 1) and it is difficult to find other prospective epidemiological studies that can be used for comparison. A study in Norway identified all children born in one city (Oslo) who had a ‘progressive childhood encephalopathy’ and found that the overall cumulative incidence of progressive childhood encephalopathy among 138 550 live births was 0.6 per 1000.^19^ The PIND study found a lifetime risk at birth of 0.1 per 1000 live births – a similar calculation to that used for the Oslo study. The lower PIND Study rate may be partly explained by the PIND case definition, which would have excluded some cases included in the Norwegian study. Also, 531 PIND Study cases were not eligible for estimating lifetime risk because they were still under investigation (*n*=284) or had been investigated without a diagnosis being made (*n*=247).

### Distribution of diseases by age and ethnicity

A previous paper generated by the PIND Study reported on the distribution of diseases found in notified children;^20^ these cases were not divided according to age or ethnic group. A separate paper reported that in some parts of the UK there were relatively large numbers of PIND cases compared with the wider UK population, with a high proportion from consanguineous families of Pakistani origin.^5^ A more recent paper reported the relatively large numbers of British Asian children in PIND Study cases who were undiagnosed despite careful investigation; in 89% of the undiagnosed Pakistani children the parents were consanguineous.^4^

Figures 1, 2, 3, 4 show considerable differences in the differential diagnosis of PIND according to age. Some disorders have been grouped to simplify the picture. In the first year of life, the mitochondrial disorders made up the largest group. Mitochondrial diseases often present with a relatively non‐specific picture,^15^ so it is important to consider them in young infants with PIND. Children with PIND mainly have genetically determined neurometabolic or neurodegenerative diseases, which present early in life (81% of the PIND cases developed symptoms or signs before 5y of age). The Oslo study also found a strong reduction in incidence rates with increasing age.^19^

This paper reports the differences in the distribution of diseases in the two biggest ethnic groups – White and Pakistani (58.2% and 17% of the diagnosed cases respectively). Of the 340 Pakistani children, 86% had consanguineous parents. The Pakistani group included many different families with a wide spectrum of disorders. There were some differences between White and Pakistani children, for instance, there was no case of Menkes disease in the Pakistani children. However, there was considerable similarity in the patterns of disease found in these two groups of children from very different ethnic backgrounds.

## Conclusions

The diagnosis of vCJD can only be confirmed or excluded with certainty by neuropathological study. If ante‐mortem brain biopsies or post‐mortem studies of brain tissue were always performed in children with undiagnosed neurodegenerative diseases, then vCJD would be detected and there would be no need for the PIND Study. However, we have previously reported that the undiagnosed children in the PIND Study rarely underwent ante‐ or post‐mortem pathological examination of brain tissue^4^ and this seems unlikely to change. Therefore, in the absence of an alternative validated vCJD screening test, the PIND Study remains the only practical means of performing systematic surveillance for vCJD in children in the UK. The aim of this paper is to help paediatricians by providing unique data about the age‐related differential diagnosis in children presenting with PIND.

## Supporting information


**Table S1:** White and Pakistani children aged <1 year not included in Figure 1

**Table S2:** White and Pakistani children aged 1 to 4 years not included in Figure 2

**Table S3:** White and Pakistani children aged 5 to 9 years not included in Figure 3

**Table S4:** White children aged 10 to 15 years not included in Figure 4
