# Supplementary material for: Differential diagnosis of progressive intellectual and neurological deterioration in children
Source: Dev Med Child Neurol. 2020 Sep 24;63(3):287–94. doi: 10.1111/dmcn.14691 (PMC7891454; doi:10.1111/dmcn.14691)
Supplement: Supplementary file 1 — Table S1: White and Pakistani children aged <1 year not included in Figure 1 [file DMCN-63-287-s001.docx]

**Table S1.** White and Pakistani children age <1 year not included in Figure 1

| **White < 1y n = 150** |  |
| --- | --- |
| Pelizaeus-Merzbacher disease | 9 |
| Canavan disease | 8 |
| Hypomyelination | 8 |
| MPS II, Hunter syndrome | 8 |
| Neuronal ceroid lipofuscinosis, infantile | 8 |
| Non ketotic hyperglycinaemia | 8 |
| Cockayne disease | 7 |
| Niemann-Pick disease type C | 7 |
| Unclassified leukoencephalopathy | 7 |
| Glutaric aciduria type 1 | 6 |
| D-bifunctional protein deficiency | 5 |
| Carbohydrate-deficient glycoprotein syndrome type 1a | 4 |
| I-Cell disease | 4 |
| MPS IIIA, SanFilippo syndrome | 4 |
| Megalencephalic leukoencephalopathy with sub cortical cysts | 3 |
| Methylmalonic aciduria | 3 |
| Cerebellar atrophy, anterior horn cell disease & dystonia (*EXOSC3* mutation) | 2 |
| Gaucher disease | 2 |
| Late infantile metachromatic leukodystrophy | 2 |
| Lesch-Nyhan disease | 2 |
| Methylenetetrahydrofolate reductase deficiency | 2 |
| Niemann-Pick disease type A | 2 |
| PKAN (NBIA) | 2 |
| Vanishing white matter disease | 2 |
| 4 hydroxybutyric aciduria (succinic semialdehyde dehydrogenase deficiency) | 1 |
| BPAN (NBIA) | 1 |
| Bruton's agammaglobulinaemia | 1 |
| Chromosome 16 duplication | 1 |
| Chronic herpes simplex encephalitis | 1 |
| Citrullinaemia type 1 | 1 |
| Coats plus syndrome | 1 |
| Congenital hyperinsulinaemic hypoglycaemia | 1 |
| *CSTB* gene mutation | 1 |
| Degos disease | 1 |
| DIDMOAD | 1 |
| DRPLA | 1 |
| Fucosidosis | 1 |
| *GNAO1* encephalopathy | 1 |
| Hereditary folate malabsorption (*SLC46A1* mutation) | 1 |
| HIV encephalopathy | 1 |
| Interferonopathy- *IFIH1* gene mutation | 1 |
| *Ip36* deletion syndrome | 1 |
| L-2 hydroxyglutaric aciduria | 1 |
| *TUBB4A*-related leukodystrophy | 1 |
| *MECP2* related syndrome | 1 |
| Merosin negative congenital muscular dystrophy | 1 |
| Molybdenum co-factor deficiency | 1 |
| Neuronal ceroid lipofuscinosis, late infantile | 1 |
| Non ketotic hyperglycinaemia | 1 |
| PLAN (NBIA) | 1 |
| Pontocerebellar hypoplasia type 1 | 1 |
| Pontocerebellar hypoplasia type 2 (*TSEN54* mutation) | 1 |
| *SCN8A*-related epilepsy | 1 |
| Sialic acid storage disease | 1 |
| Spinocerebellar ataxia type 2 | 1 |
| *STXBP1* encephalopathy with epilepsy | 1 |
| Sulphite oxidase deficiency | 1 |
| Tuberous sclerosis complex | 1 |
| Ubiquinone deficiency | 1 |
|  |  |
| **Pakistani < 1 year n = 62** |  |
| Sandhoff disease | 4 |
| Hypomyelination | 4 |
| Niemann-Pick type C | 4 |
| PKAN (NBIA) | 4 |
| PLAN (NBIA) | 4 |
| MPS I, Hurler syndrome | 3 |
| Multiple sulphatase deficiency | 3 |
| Nonketotic hyperglycinaemia | 3 |
| Biotinidase deficiency | 2 |
| D-bifunctional protein deficiency | 2 |
| Glutaric aciduria type 1 | 2 |
| Sulphite oxidase deficiency | 2 |
| *WWOX* gene related encephalopathy | 2 |
| 3-hydroxyisobutytry-CoA hydrolase deficiency | 1 |
| Arginase deficiency | 1 |
| Argininosuccinic aciduria | 1 |
| *ATAD3A* gene mutation | 1 |
| Beta-mannosidosis | 1 |
| Carbohydrate deficient glycoprotein syndrome 1a | 1 |
| Cerebral folate transport deficiency | 1 |
| I-Cell disease | 1 |
| Intracellular defect of cobalamin metabolism | 1 |
| Maple syrup urine disease | 1 |
| Megalencephalic leukoencephalopathy with subcortical cysts | 1 |
| Merosin-deficient muscular dystrophy | 1 |
| MPS III, SanFilippo syndrome | 1 |
| Neuraminidase deficiency | 1 |
| Niemann-Pick disease type A | 1 |
| Pelizaeus-Merzbacher disease | 1 |
| Rett syndrome | 1 |
| *SCN1A* mutation | 1 |
| Sialidosis type II | 1 |
| Succinic semialdehyde dehydrogenase deficiency | 1 |
| Tetrahydrobiopterin deficiency | 1 |
| Triosephosphate isomerase deficiency | 1 |
| UNC80 deficiency | 1 |

**BPAN**: beta-propeller protein-associated neurodegeneration, **DIDMOAD**: diabetes insipidus, diabetes mellitus, optic atrophy and deafness, **DRPLA**: dentatorubral-pallidoluysian atrophy, **PKAN**: pantothenate kinase-associated neurodegeneration, **PLAN**: PLA2G6-associated neurodegeneration.
