# Supplementary material for: Differential diagnosis of progressive intellectual and neurological deterioration in children
Source: Dev Med Child Neurol. 2020 Sep 24;63(3):287–94. doi: 10.1111/dmcn.14691 (PMC7891454; doi:10.1111/dmcn.14691)
Supplement: Supplementary file 2 — Table S2: White and Pakistani children aged 1 to 4 years not included in Figure 2 [file DMCN-63-287-s002.docx]

**Table S2**. White and Pakistani age 1-4 years not included in Figure 2

| **White 1-4 years n = 96** |  |
| --- | --- |
| Krabbe disease | 10 |
| Unclassified leukoencephalopathy | 8 |
| Ataxia-telangiectasia | 7 |
| MPS II, Hunter syndrome | 7 |
| PKAN (NBIA) | 6 |
| Aicardi-Goutières syndrome | 5 |
| Alexander disease | 5 |
| GM1 gangliosidosis | 5 |
| Hypomyelination | 5 |
| Glutaric aciduria type 1 | 3 |
| Huntington disease | 3 |
| L-2-hydroxyglutaric aciduria | 3 |
| Cockayne disease | 2 |
| Fucosidosis | 2 |
| Homocystinuria | 2 |
| Pelizaeus-Merzbacher disease | 2 |
| *UBTF* gene mutation | 2 |
| Carbohydrate-deficient glycoprotein syndrome type 1a | 1 |
| Chromosome 11q deletion | 1 |
| CINCA syndrome | 1 |
| CRMCC | 1 |
| DIDMOAD | 1 |
| DRPLA | 1 |
| Giant axonal neuropathy | 1 |
| Sandhoff disease | 1 |
| Lesch-Nyhan disease | 1 |
| Mannosidosis | 1 |
| MPS I, Hurler syndrome | 1 |
| Mucopolysaccharidosis (unspecified) | 1 |
| Multiple sclerosis (atypical) | 1 |
| Multiple sulphatase deficiency | 1 |
| Nonketotic hyperglycinaemia | 1 |
| PEHO (Progressive encephalopathy, oedema, hypsarrhythmia and optic atrophy) | 1 |
| SSPE | 1 |
| Temporal lobectomy for gliosis | 1 |
| Tolmie-Browne-McGettrick-Stephenson syndrome | 1 |
|  |  |
| **Pakistani 1-4 years n = 37** |  |
| Biotinidase deficiency | 3 |
| GM1 gangliosidosis | 3 |
| Mucolipidosis type IV | 3 |
| Aicardi-Goutières syndrome | 2 |
| Arginase deficiency | 2 |
| D-2-hydroxyglutaric aciduria | 2 |
| Hereditary spastic paraplegia variant with leukodystrophy | 2 |
| Hypomyelinating leukodystrophy type 11 | 2 |
| Rett syndrome | 2 |
| Succinic semialdehyde dehydrogenase deficiency | 2 |
| Adrenoleukodystrophy | 1 |
| *ATAD3A* gene mutation | 1 |
| Ataxia-telangiectasia | 1 |
| BPAN (NBIA) | 1 |
| Gaucher disease | 1 |
| Giant axonal neuropathy | 1 |
| Juvenile Alexander disease | 1 |
| Krabbe disease | 1 |
| Methylenetetrahydrofolate reductase deficiency | 1 |
| MPS II, Hunter syndrome | 1 |
| Multiple sulphatase deficiency | 1 |
| Pelizaeus-Merzbacher disease | 1 |
| Peroxisomal beta oxidation disorder | 1 |
| Vanishing white matter disease (*E1F2B* mutation) | 1 |

**BPAN**: beta-propeller protein-associated neurodegeneration, **CINCA**: chronic infantile neurological cutaneous and articular syndrome, **CRMCC**: cerebroretinal microangiopathy with calcifications and cysts, **DIDMOAD**: diabetes insipidus, diabetes mellitus, optic atrophy and deafness, **DRPLA**: dentatorubral-pallidoluysian atrophy, **PKAN**: pantothenate kinase-associated neurodegeneration, **SSPE**: subacute sclerosing panencephalitis.
