# Supplementary material for: Differential diagnosis of progressive intellectual and neurological deterioration in children
Source: Dev Med Child Neurol. 2020 Sep 24;63(3):287–94. doi: 10.1111/dmcn.14691 (PMC7891454; doi:10.1111/dmcn.14691)
Supplement: Supplementary file 3 — Table S3: White and Pakistani children aged 5 to 9 years not included in Figure 3 [file DMCN-63-287-s003.docx]

**Table S3**. White and Pakistani age 5-9 years not included in Figure 3

| **White 5-9 years n = 18** |  |
| --- | --- |
| MPS III, SanFilippo syndrome | 2 |
| Rett syndrome | 2 |
| Vanishing white matter disease | 2 |
| Aicardi-Goutières syndrome with nigrostriatal necrosis (*ADAR1* mutation) | 1 |
| ALL with post-radiation CNS relapse | 1 |
| Ataxia-telangiectasia | 1 |
| LBSL (*DARS2* mutation) | 1 |
| DIDMOAD | 1 |
| Episodic ataxia Type II | 1 |
| Krabbe disease | 1 |
| L-2-hydroxyglutaric aciduria | 1 |
| Langerhans histiocytosis with neurological deficit | 1 |
| Medulloblastoma | 1 |
| Rett, atypical syndrome | 1 |
| Unclassified leukoencephalopathy | 1 |
|  |  |
| **Pakistani 5-9 years n = 10** |  |
| Adrenoleukodystrophy | 1 |
| Argininosuccinic aciduria | 1 |
| *ATAD3A* gene deletion | 1 |
| Ataxia-telangiectasia | 1 |
| CADASIL | 1 |
| Infantile neuroaxonal dystrophy | 1 |
| MPS III, SanFillipo syndrome | 1 |
| Niemann-Pick type C | 1 |
| Rasmussen encephalitis | 1 |
| SSPE | 1 |

**CADASIL**: cerebral autosomal dominant arteriopathy with subcortical infarcts and leukoencephalopathy, **DIDMOAD**: diabetes insipidus, diabetes mellitus, optic atrophy and deafness, **LBSL**: leukoencephalopathy with brainstem and spinal cord involvement and lactic acidosis, **SSPE**: subacute sclerosing panencephalitis.
