# Supplementary material for: Differential diagnosis of progressive intellectual and neurological deterioration in children
Source: Dev Med Child Neurol. 2020 Sep 24;63(3):287–94. doi: 10.1111/dmcn.14691 (PMC7891454; doi:10.1111/dmcn.14691)
Supplement: Supplementary file 4 — Table S4: White children aged 10 to 15 years not included in Figure 4 [file DMCN-63-287-s004.docx]

**Table S4.** White age 10-15 years not included in Figure 4

| **White 10-15 years n = 12** |  |
| --- | --- |
| Astrocytoma | 1 |
| Carbohydrate-deficient glycoprotein syndrome type 1a | 1 |
| CLIPPERS | 1 |
| Cockayne disease | 1 |
| DIDMOAD | 1 |
| DRPLA | 1 |
| GM1 gangliosidosis, Juvenile | 1 |
| Hypomyelination | 1 |
| Immunodefective encephalopathy | 1 |
| LCC (Labrune syndrome) | 1 |
| LBSL (*DARS2* mutation) | 1 |
| Vanishing white matter disease | 1 |

**CLIPPERS**: chronic lymphocytic inflammation with pontine perivascular enhancement responsive to steroids, **DIDMOAD**: diabetes insipidus, diabetes mellitus, optic atrophy and deafness, **DRPLA**: dentatorubral-pallidoluysian atrophy, **LCC**: leukoencephalopathy intracranial calcifications and cysts, **LBSL**: leukoencephalopathy with brainstem and spinal cord involvement and lactic acidosis.
